# Risk prediction model for major adverse cardiovascular events (MACE) during hospitalization in patients with coronary heart disease based on myocardial energy metabolic substrate

**DOI:** 10.3389/fcvm.2023.1137778

**Published:** 2023-05-03

**Authors:** Li Na, Jia Lin, Yao Kuiwu

**Affiliations:** ^1^ Department of Cardiology, Guang’anmen Hospital, China Academy of Chinese Medical Sciences, Beijing, China; ^2^ Department of Artillery Engineering, Army Engineering University of PLA, Shijiazhuang, China; ^3^ Department of Medicine, Eye Hospital China Academy of Chinese Medical Sciences, Beijing, China

**Keywords:** coronary heart disease, myocardial energy metabolic substrate, nomogram, risk prediction model, MACE

## Abstract

**Background:**

The early attack of coronary heart disease (CHD) is very hidden, and clinical symptoms generally do not appear until cardiovascular events occur. Therefore, an innovative method is needed to judge the risk of cardiovascular events and guide clinical decision conveniently and sensitively. The purpose of this study is to find out the risk factors related to MACE during hospitalization. In order to develop and verify the prediction model of energy metabolism substrates, and establish a nomogram to predict the incidence of MACE during hospitalization and evaluate their performance.

**Methods:**

The data were collected from the medical record data of Guang'anmen Hospital. This review study was collected the comprehensive clinical data of 5,935 adult patients hospitalized in the cardiovascular department from 2016 to 2021. The outcome index was the MACE during hospitalization. According to the occurrence of MACE during hospitalization, these data were divided into MACE group (*n* = 2,603) and non-MACE group (*n* = 425). Logistic regression was used to screen risk factors, and establish the nomogram to predict the risk of MACE during hospitalization. Calibration curve, C index and decision curve were used to evaluate the prediction model, and drawn ROC curve to find the best boundary value of risk factors.

**Results:**

The logistic regression model was used to establish a risk model. Univariate logistic regression model was mainly used to screen the factors significantly related to MACE during hospitalization in the training set (each variable is put into the model in turn). According to the factors with statistical significance in univariate logistic regression, five cardiac energy metabolism risk factors, including age, albumin(ALB), free fatty acid(FFA), glucose(GLU) and apolipoprotein A1(ApoA1), were finally input into the multivariate logistic regression model as the risk model, and their nomogram were drawn. The sample size of the training set was 2,120, the sample size of the validation set was 908. The C index of the training set is 0.655 [0.621,0.689], and the C index of the validation set was 0.674 [0.623,0.724]. The calibration curve and clinical decision curve show that the model performs well. The ROC curve was used to establish the best boundary value of the five risk factors, which could quantitatively present the changes of cardiac energy metabolism substrate, and finally achieved prediction of MACE during hospitalization conveniently and sensitively.

**Conclusion:**

Age, albumin, free fatty acid, glucose and apolipoprotein A1 are independent factors of CHD in MACE during hospitalization. The nomogram based on the above factors of myocardial energy metabolism substrate provides prognosis prediction accurately.

## 1. Introduction

Coronary heart disease (CHD) is a major global public health problem, which seriously affects the quality of life of human beings, and brings economic and health burdens to patients and their family. The early attack of CHD is very hidden, and clinical symptoms generally do not appear until cardiovascular events occur.The number of inpatients with ST segment elevation myocardial infarction increased significantly from 2001 to 2011 (1), but the risk of death during hospitalization did not decrease during the same period (2). Therefore, it is very important to evaluate quickly and accurately, and communicate with patients and their families in a timely and effective manner.

Metabolism is the basic requirement of the body. Fatty acids used by myocardium account for 25%–63% of the total substrate, glucose 16%–31%, ketone body 5%–61%, and amino acids 5.6%. Fatty acid is the main substrate of adult myocardial energy supply, but the glycolysis is the main way of energy supply in the early stage of acute ischemia and hypoxia. The glucose substrate is limited and ATP produced by anaerobic glycolysis is limited. When myocardial ischemia and hypoxia are not alleviated, ATP compensation will be insufficient, and ketone body, fatty acid, amino acid metabolism and other methods will be started at this time.After myocardial infarction, the myocardium will undergo irreversible ultrastructural changes. The decrease of ATP stimulates the mitochondrial oxidation function, causing the increase of coronary blood flow to provide more oxygen for the myocardium. Moreover, ATP reduced products such as ADP, AMP and other cleavage products can further enhance mitochondrial oxidative phosphorylation as regulatory factors. The optimal cardiac energy supply depends on the balanced utilization of substrates (such as fatty acids and glucose). The research shows that an intervention to rebalance fuel supply is used to manipulate the uptake of cell substrates, in order to help restore damaged organ functions. Therefore, the change of energy supply substrates is crucial to the prognosis of the heart (3).

Under acute ischemia and hypoxia, glucose metabolism characterized by low oxygen consumption is increased to meet the ATP demands of the heart. However, it inhibits the energy supply of fatty acids, causing the accumulation of lipids and inducing lipotoxicity. Studies have shown that lipid metabolism toxicity is an independent risk factor for the aggravation of CHD, and the increase of free fatty acid (FFA) level is an independent risk factor and independent diagnostic marker of acute myocardial infarction (4). The changes of metabolic status and lipid deposition of vascular wall in patients with coronary heart disease lead to endothelial cell dysfunction, macrophage activation and plaque instability, which ultimately lead to adverse cardiovascular events. Research shows that age has a significant impact on myocardial infarction and is a risk factor for atherosclerosis (5). ApoAl is the main apolipoprotein of high-density lipoprotein (HDL-C). ApoAl can transport cholesterol from the body tissues to the liver for catabolism, thus preventing cholesterol from depositing in the blood vessel wall. Therefore, ApoAl is an anti atherosclerotic indicator *in vivo*, which can inhibit the formation of atherosclerotic plaque. The study confirmed that ApoA1 is superior to HDL-C in evaluating the severity of coronary artery injury (6). In addition, studies have shown that the level of ALB and GLU at admission is an independent predictor of cardiovascular death in patients with myocardial infarction (7–9). Therefore, the risk indicators of FFA, ApoA1, GLU, age and ALB selected in this study have clinical significance.

The risk prediction of coronary heart disease is highly recommended in the clinical practice guidelines in Europe, America and China (10). Many models have been established, but the complexity of model operation makes it difficult to collect indicators in a short time. The purpose of our research is to establish a simple and easily accessible risk model of CHD. Metabolic disorder is one of the causes of CHD, but myocardial energy metabolic substrate have not been reflected in the existing models. This study found that five risk factors related to myocardial energy metabolismsubstrate had significant statistical significance on MACE, so these indicators were included in the prediction model of CHD. According to the above results, the nomogram was used to analyze the prognostic effect of energy metabolism substrate on patients, and the best boundary value of predictive factors was obtained through ROC analysis, and the predictive model was presented quantitatively. The purpose of our study is to develop a simple and feasible risk model to predict the MACE during hospitalization of CHD, and verify its performance in the Chinese patient population.

## 2. Materials and methods

### 2.1. Study population

These data were retrieved from the medical record data of Guang'anmen Hospital. The database integrated the comprehensive clinical data of 5,935 adult patients hospitalized in the cardiovascular department from 2016 to 2021. The inclusion criteria were those patients diagnosed as coronary heart disease who were older than 18 years old. The overall data was saved as a relational database, including patient demographics, laboratory examinations, and hospital MACE. The use of the data set of the medical record data of Guang'anmen Hospital had been approved by the Ethics Committee. In order to protect the privacy of patients, all patients in the database had been removed from identification and do not need informed consent. This study was conducted in accordance with the recommendations of the Transparent Reporting of a multivariable prediction model for Individual Prognosis Or Diagnosis statement (11).

### 2.2. Study cohort

Patients admitted to the cardiovascular department of Guang'anmen Hospital who were diagnosed with CHD were eligible for inclusion. 5,935 patients with CHD were included for analysis. The queue was randomly divided into training set and verification set at a ratio of 7:3. The training set was used to establish the nomogram, and the verification set was used to verify the regression model. The sample size of the training set was 2,120, and the sample size of the validation set was 908.

### 2.3. Data extraction

Structured query language was used for data extraction. All data regarding baseline characteristics were collected as the first value in the initial 24 h following admission. The variables analyzed included (1) basic demographic statistics, including age, sex, smoking and drinking history; (2) Vital signs, including heart rate and blood pressure; (3) Laboratory examination, including C-reactive protein, D-dimer, lactate dehydrogenase, albumin, total protein, white ratio, low-density lipoprotein, triglyceride, high-density lipoprotein, very low-density lipoprotein, total cholesterol, lipoprotein (a), high-sensitivity C-reactive protein, apolipoprotein A1, apolipoprotein B, glucose, lactic acid, free fatty acid, total bile acid, homocysteine; (4) Killip cardiac function class. In this study, we used MACE during hospitalization as an outcome indicator, which was also extracted from the database.

The MACE during hospitalization was used as the outcome indicator. The MACE refers to heart failure, severe arrhythmia (persistent ventricular tachycardia, ventricular fibrillation, new hemodynamic instability AF or atrial flutter, high-grade atrioventricular block, excluding reperfusion arrhythmia during PCI), angina pectoris after myocardial infarction and death, acute myocardial infarction, ischemic stroke, peripheral artery occlusion, recurrent angina, and cardiac death. All information that might indicate MACE was further investigated by examining the hospital medical records or general practitioner. Then, two cardiologists would independently determine all potential events to determine whether MACE occurs.

### 2.4. Management of missing data

In this review study, cases of patients without medical history information or partial test results were excluded.

### 2.5. Statistical analysis

SPSS 22.0 and R 4.0.3 statistical software were used for statistical analysis. Continuous (quantitative) data were expressed by mean ± standard deviation. Continuous variables were compared using Student's *t*-test or wilcox test between the two groups, as appropriate. Classified (qualitative) data were expressed by frequency (percentage) and compared between groups by *χ*^2^ test or Fisher's exact test. The difference was considered statistically significant when *P* value < 0.05.

In this study, the goal was to develop a rapid prognostic model for MACE with CHD. The data were divided into MACE group and non-MACE group during hospitalization, and the variables with statistical difference between the two groups were compared. The logistic regression model was used to establish the risk model by the glm function, the univariate logistic regression model was mainly used to screen the factors significantly in the training set, and the potential risk factors in the univariate logistic regression were input into the multivariate logistic regression model. Multivariate logistic model determines risk factors, and then assembles them into nomograms to predict MACE during hospitalization. The continuous variables were replaced by the binary variable, and the Logistic regression analysis was carried out. The ROC curve was used to select the maximum value of the Jordan index as the best boundary value. After establishing the model, the predictive ability of the prediction model was evaluated by using calibration curve, clinical decision curve, and C index.

## 3. Results

### 3.1. Comparison of clinical data between MACE group and non-MACE patients

The patient characteristics were summarized as follows: Table 1 collects the hospital medical records with MACE events (*n* = 2,603) and non-MACE events (*n* = 425). 1,485 (49.04%) of 3,028 patients were male, the average age was 72 years old, and the heart rate was 76 beats per minute. There was no significant difference in sex, heart rate, blood pressure, smoking, drinking history, D-dimer, triglyceride, high-density lipoprotein, very low density lipoprotein, lipoprotein (a), apolipoprotein B, total bile acid, and serum homocysteine between MACE group and non-MACE group. The comparison of each variable were shown in Table 1.

**Table 1:** Comparison of general clinical data between major adverse cardiovascular events (MACE) group and non-MACE group.

Variables	Non-MACE group (*n* = 2,603)	MACE group (*n* = 425)	Sum (*n* = 3,028)	*P*-value
**General demographics data**				
Male, *n* (%)	1,255 (48.21%)	230 (54.12%)	1,485 (49.04%)	0.443
Age, years (24–97)	72 ± 11.82	75.64 ± 10.97	72.51 ± 11.77	<0.001
Killip grade II–IV, *n* (%)				<0.001
I	167 (10.17%)	25 (9.33%)	192 (10.05%)	
II	758 (46.16%)	98 (36.57%)	856 (44.82%)	
III	534 (32.52%)	86 (32.09%)	620 (32.46%)	
IV	183 (11.14%)	59 (22.01%)	242 (12.67%)	
Heart rate, beats/minute (17–156)	75.87 ± 14.22	77.07 ± 16.75	76.03 ± 14.6	0.246
Systolic blood pressure, mmHg (70–236)	137.18 ± 20.19	136.44 ± 21.25	137.07 ± 20.34	0.562
Diastolic blood pressure, mmHg (32–137)	74.37 ± 12.36	72.69 ± 13.2	74.14 ± 12.49	0.03
Smoking, *n* (%)				0.881
No	1,926 (73.99%)	313 (73.65%)	2,239 (73.94%)	
Yes	677 (26.01%)	112 (26.35%)	789 (26.06%)	
Drinking, *n* (%)				0.496
No	2,234 (85.82%)	370 (87.06%)	2,604 (86%)	
Yes	369 (14.18%)	55 (12.94%)	424 (14%)	
**Laboratory tests**				
C-reactive protein, mg/L (0.5–193.15)	10.56 ± 23.45	18.61 ± 32.05	11.72 ± 25.04	<0.001
D dimer, mg/L (0.15–35)	0.52 ± 0.51	3.7 ± 3.56	1.11 ± 1.94	0.116
Lactate dehydrogenase, U/L (87–2,938)	193.82 ± 71.15	259.96 ± 283.84	203.11 ± 127.15	<0.001
Albumin, g/L (21.6–54.2)	39.42 ± 4.84	36.85 ± 5.45	39.06 ± 5.01	<0.001
Total protein, g/L (42.5–86.8)	66.46 ± 6.39	64.01 ± 6.77	66.11 ± 6.5	<0.001
Albumin/Globulin (0.52–3.2)	1.5 ± 0.32	1.4 ± 0.32	1.49 ± 0.32	<0.001
Low-density lipoprotein cholesterol C, mmol/L (0.64–6.99)	2.52 ± 0.81	2.4 ± 0.79	2.5 ± 0.81	0.004
Triglycerides,mmol/L (0.14–20.74)	1.51 ± 0.92	1.48 ± 1.31	1.5 ± 0.98	0.563
High-density lipoprotein cholesterol C, mmol/L (0.28–2.99)	1.08 ± 0.28	1.05 ± 0.31	1.08 ± 0.29	0.026
Very low-density lipoprotein, mmol/L (0.06–9.43)	0.69 ± 0.42	0.67 ± 0.6	0.68 ± 0.45	0.559
Total cholesterol, mmol/L (1.21–14.83)	3.93 ± 1.08	3.74 ± 1.11	3.9 ± 1.08	0.001
Lipoprotein(a),mg/dl (0.09–176.8)	18.62 ± 20.63	19.82 ± 23.2	18.79 ± 21.01	0.275
Highly sensitive C-reactive protein, mg/l (0.08–210.02)	9.77 ± 24.09	20.51 ± 36.1	11.14 ± 26.16	<0.001
Apolipoprotein A1, g/L (0.34–2.43)	1.16 ± 0.24	1.08 ± 0.24	1.14 ± 0.24	<0.001
Apolipoprotein B, g/L (0.25–2.23)	0.75 ± 0.22	0.72 ± 0.21	0.75 ± 0.22	0.015
Glucose, mmol/L (0.64–33.4)	7.74 ± 3.45	8.42 ± 3.82	7.83 ± 3.51	0.001
Lactic acid, mmol/L (0.45–24.56)	2.09 ± 0.75	2.63 ± 2.47	2.2 ± 1.33	0.01
Free fatty acid, umol/L (0.03–2.35)	0.55 ± 0.28	0.62 ± 0.31	0.56 ± 0.29	<0.001
Total bile acids, μmol/L (0.2–252.9)	7.22 ± 10.45	7.94 ± 11.32	7.32 ± 10.58	0.217
Serum homocysteine, μmol/L (4.47–67.4)	15.7 ± 7.23	16.71 ± 7.54	15.83 ± 7.28	0.03

The results were expressed as mean ± standard deviation, or percentage, *P* < 0.05 was statistically significant.

### 3.2. Baseline characteristics of the training set and validation set

The training set and verification set consisted of 2,120 and 908 CHD patients, respectively. The baseline characteristics were shown in Table 2. There were no significant differences in each risk factors between training set and verification set (all *P* > 0.05).

**Table 2:** Comparison of basic demographics, vital signs, laboratory tests and the MACE during hospitalization between training set and validation set.

Variables	Training set (*n* = 2,120)	Validation set (*n* = 908)	Sum (*n* = 3,028)	*P*-value
Male, *n*(%)	1,048 (49.43%)	437 (48.13%)	1,485 (49.04%)	0.510
Age, years	72.42 ± 12.04	72.72 ± 11.12	72.51 ± 11.77	0.517
Killip grade II–IV, *n* (%)				0.076
I	138 (10.42%)	54 (9.23%)	192 (10.05%)	
II	589 (44.45%)	267 (45.64%)	856 (44.82%)	
III	415 (31.32%)	205 (35.04%)	620 (32.46%)	
IV	183 (13.81%)	59 (10.09%)	242 (12.67%)	
Heart rate, beats/minute	76.42 ± 14.63	75.17 ± 14.51	76.03 ± 14.6	0.071
Systolic blood pressure, mmHg	137.31 ± 20.55	136.54 ± 19.86	137.07 ± 20.34	0.421
Diastolic blood pressure, mmHg	74.21 ± 12.72	73.97 ± 11.98	74.14 ± 12.49	0.675
Smoking, *n* (%)				0.493
No	1,560 (73.58%)	679 (74.78%)	2,239 (73.94%)	
Yes	560 (26.42%)	229 (25.22%)	789 (26.06%)	
Drinking, *n* (%)				0.106
No	1,809 (85.33%)	795 (87.56%)	2,604 (86%)	
Yes	311 (14.67%)	113 (12.44%)	424 (14%)	
C-reactive protein, mg/L	11.69 ± 25.57	11.79 ± 23.74	11.72 ± 25.04	0.926
D dimer, mg/L	0.98 ± 1.39	1.36 ± 2.83	1.11 ± 1.94	0.637
Albumin, g/L	39.02 ± 5.05	39.15 ± 4.93	39.06 ± 5.01	0.517
Albumin/Globulin	1.49 ± 0.32	1.48 ± 0.33	1.49 ± 0.32	0.741
Low-density lipoprotein cholesterol C, mmol/L	2.5 ± 0.81	2.5 ± 0.8	2.5 ± 0.81	0.932
Triglycerides, mmol/L	1.48 ± 0.89	1.56 ± 1.17	1.5 ± 0.98	0.070
High-density lipoprotein cholesterol C, mmol/L	1.08 ± 0.29	1.06 ± 0.28	1.08 ± 0.29	0.126
Highly sensitive C-reactive protein, mg/L	11.05 ± 25.81	11.34 ± 26.98	11.14 ± 26.16	0.802
Very low-density lipoprotein, mmol/L	0.67 ± 0.4	0.71 ± 0.53	0.68 ± 0.45	0.069
Glucose, mmol/L	7.82 ± 3.49	7.87 ± 3.56	7.83 ± 3.51	0.728
Lactic acid, mmol/L	2.21 ± 1.42	2.2 ± 1.12	2.2 ± 1.33	0.920
Lactate dehydrogenase, U/L	205.44 ± 131.26	197.66 ± 116.87	203.11 ± 127.15	0.107
Serum homocysteine, μmol/L	15.95 ± 7.43	15.53 ± 6.9	15.83 ± 7.28	0.207
Free fatty acid, umol/L	0.57 ± 0.29	0.54 ± 0.28	0.56 ± 0.29	0.064
Apolipoprotein A1, mg/dl	1.14 ± 0.24	1.15 ± 0.24	1.14 ± 0.24	0.403
Apolipoprotein B, g/L	0.74 ± 0.22	0.75 ± 0.21	0.75 ± 0.22	0.409
Lipoprotein(a), mg/dl	18.39 ± 20.75	19.73 ± 21.58	18.79 ± 21.01	0.109
Total cholesterol, mmol/L	3.9 ± 1.07	3.92 ± 1.11	3.9 ± 1.08	0.592
Total bile acids, μmol/L	7.37 ± 11.22	7.21 ± 8.9	7.32 ± 10.58	0.713
Total protein, g/L	66.02 ± 6.65	66.34 ± 6.13	66.11 ± 6.5	0.207
Major adverse cardiovascular events(MACE) during hospitalization, *n* (%)				0.770
No	1,825 (86.08%)	778 (85.68%)	2,603 (85.96%)	
Yes	295 (13.92%)	130 (14.32%)	425 (14.04%)	

The results were expressed as mean ± standard deviation, or percentage, *P* < 0.05 was statistically significant.

### 3.3. Univariate and multivariate analysis between risk factors and the MACE during hospitalization

The univariate logistic regression analysis showed that age, C-reactive protein, albumin, high-sensitivity C-reactive protein, glucose, lactic acid, lactate dehydrogenase, free fatty acid, apolipoprotein A1 (*P* < 0.05). The potential risk factors in the screened univariate Logistic regression model were input into the multivariable Logistic regression model, and the multivariable Logistics risk model (including age, FFA, ApoA1, ALB, GLU) (*P* < 0.01) was obtained. It was found that the prediction model of myocardial energy substrate, such as albumin, glucose, and lipid composition could predict MACE during hospitalization well. As shown in Table 3.

**Table 3:** Univariate and multivariate analysis of the relationship between candidate risk factors and the MACE during hospitalization.

	Univariate logical analysis		Multivariate logical analysis	
Variables	OR [95%CI]	*P*-value	OR [95%CI]	*P*-value
Age, years	1.02817 [1.02089, 1.03551]	<0.001	1.02 [1.01, 1.03]	0.001
C-reactive protein, mg/L	1.01063 [1.00794, 1.01332]	<0.001		
Albumin, g/L	0.90258 [0.88826, 0.91713]	<0.001	0.93 [0.91, 0.95]	<0.001
Highly sensitive C-reactive protein, mg/L	1.01167 [1.00891, 1.01444]	<0.001		
Glucose, mmol/L	1.05791 [1.03637, 1.07991]	<0.001	1.04 [1.02, 1.07]	0.002
Lactic acid, mmol/L	1.34523 [1.22555, 1.4766]	<0.001		
Lactate dehydrogenase, U/L	1.00278 [1.00211, 1.00346]	<0.001		
Free fatty acid, umol/L	2.1483 [1.55366, 2.97053]	<0.001	1.67 [1.19, 2.35]	0.003
Apolipoprotein A1, mg/dl	0.19704 [0.13926, 0.2788]	<0.001	0.44 [0.28, 0.71]	0.001

The results were expressed as mean ± standard deviation, or percentage, *P* < 0.05 was statistically significant.

### 3.4. Performance evaluation of the prognostic nomogram

**Figure 1:**
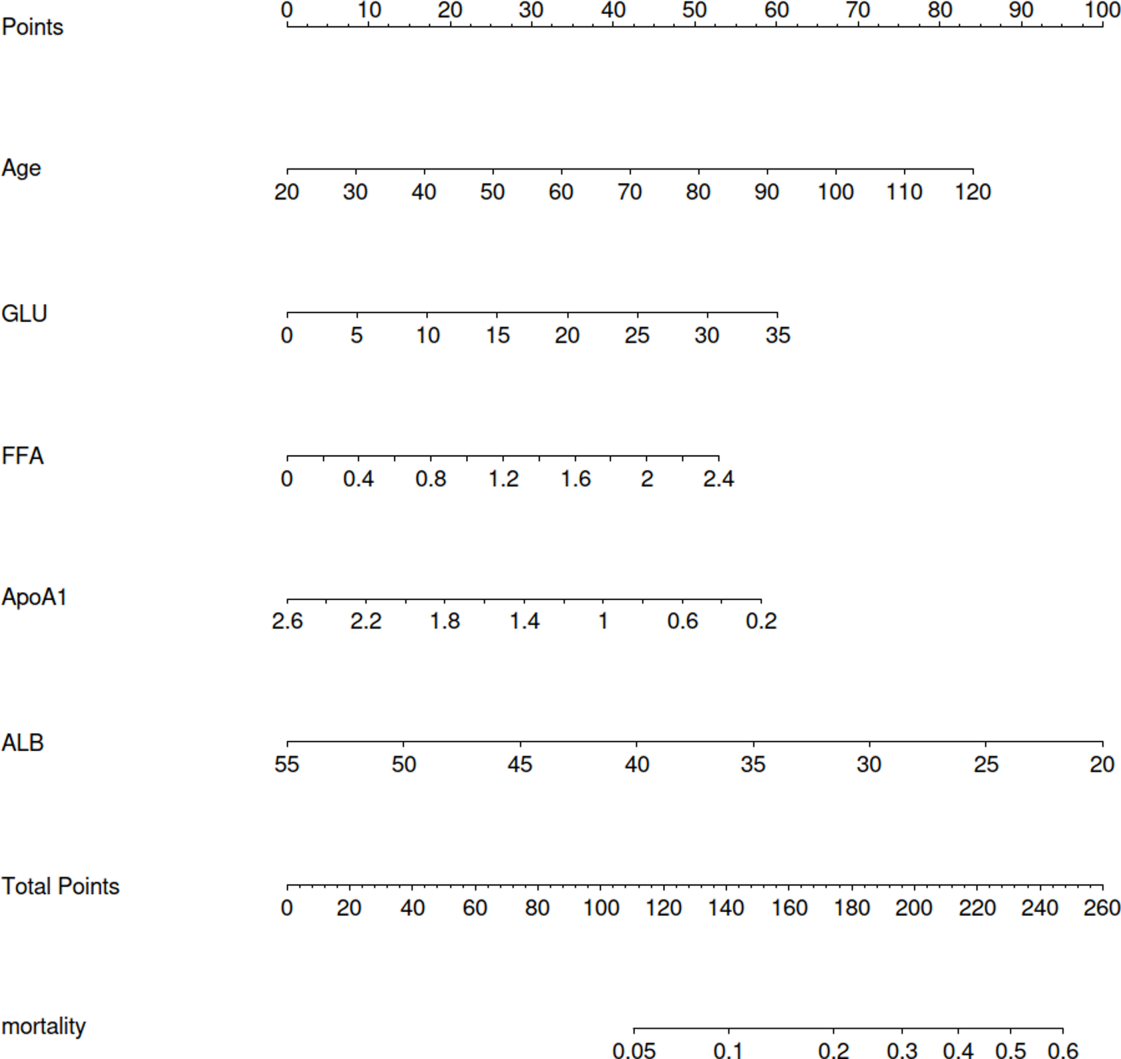
Logistic regression prognosis model nomogram.

A regression model including age, FFA, ApoA1, ALB and GLU was established according to Table 3. In order to intuitively predict the incidence of MACE during hospitalization, multivariate logistic regression model was used to draw the prognosis nomogram as shown in Figure 1. The scales of age, GLU, FFA, ApoA1 and ALB levels in the nomogram were 20–120, 0–35, 0–2.4, 2.6–0.2 and 55–20 respectively. The maximum total score was 260, and the range of in-hospital survival probability scale was 0.05–0.6.

**Figure 2:**
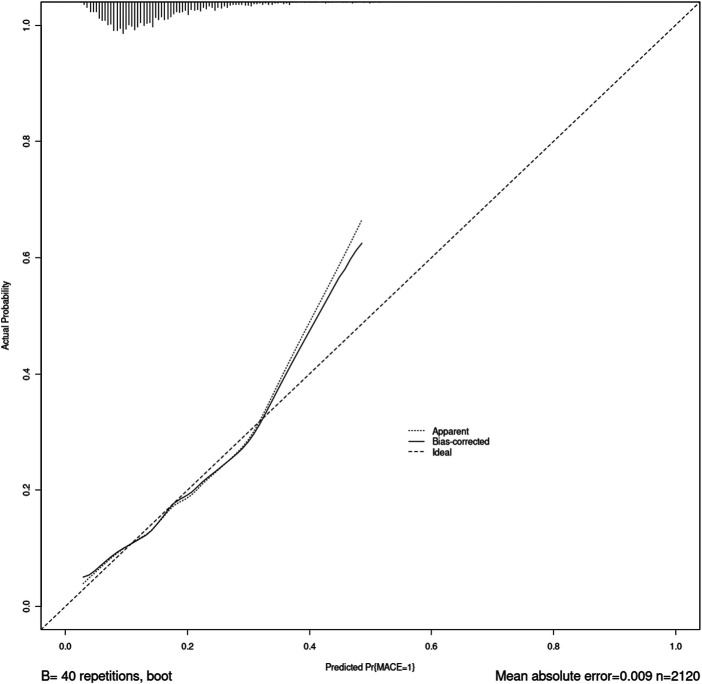
Calibration curve of subject oprating characteristics.

**Figure 3:**
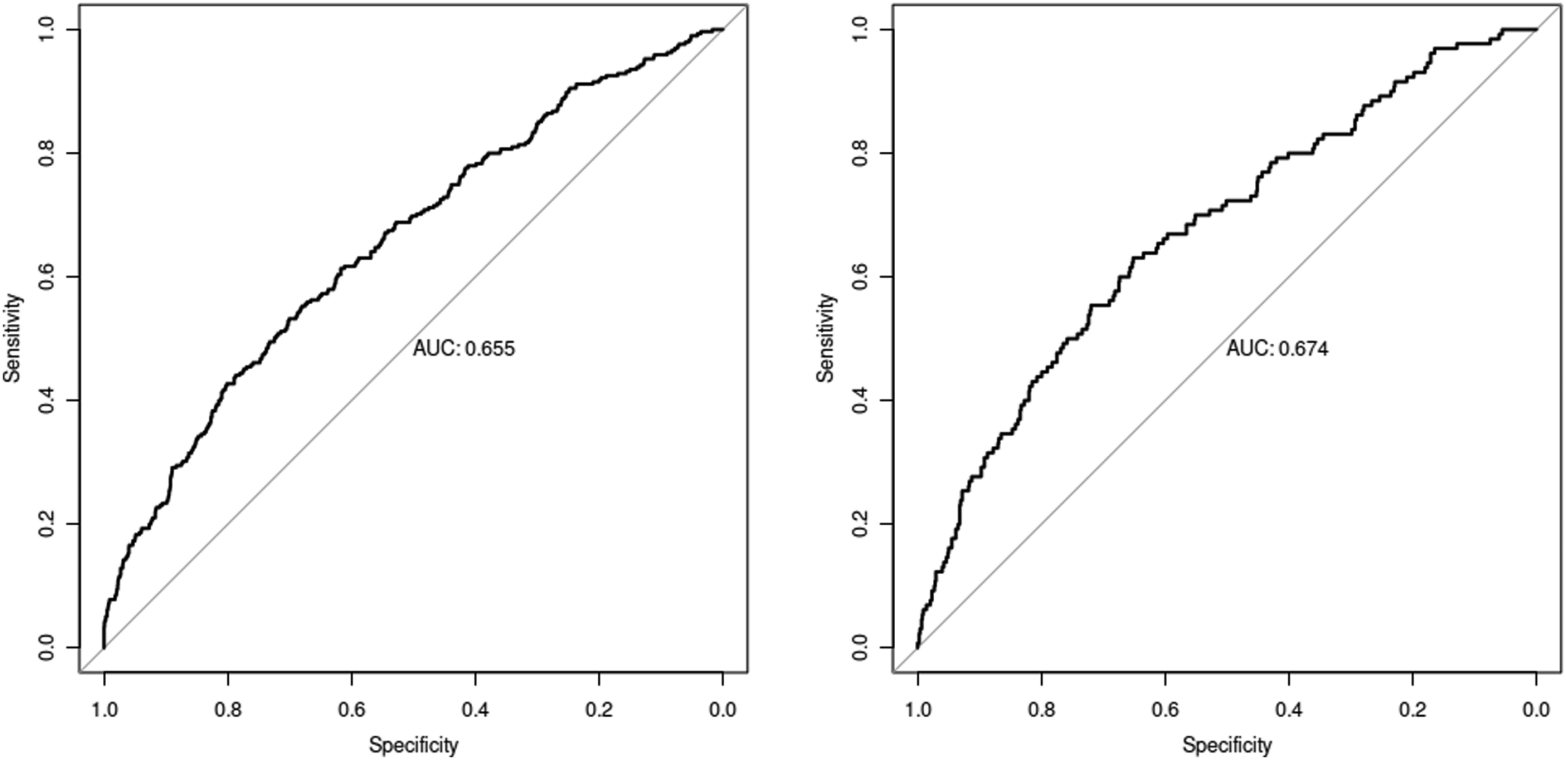
ROC curve (The training set result on the left and the validation set result on the right).

The nomogram was made from the results in Table 3 as shown in Figure 1. The higher the score calculated according to the sum of the allocations of each prognostic factor in the nomogram, the higher the probability of MACE during hospitalization. Using the data of the validation set to draw the calibration curve as shown in Figure 2. Using pROC to draw the ROC curve as shown in Figure 3. We calculated the AUC (C index) under the ROC curve of the training set as 0.655 [0.621,0.689], and the AUC (C index) under the ROC curve of the validation set as 0.674 [0.623,0.724]. In the validation set, the model was subject to Hosmer Leishow Goodness of Fit Test. The chi square value was 6.964, *P* = 0.54 (*P* > 0.05), indicating that there was no difference between the training set and the validation set.

**Figure 4:**
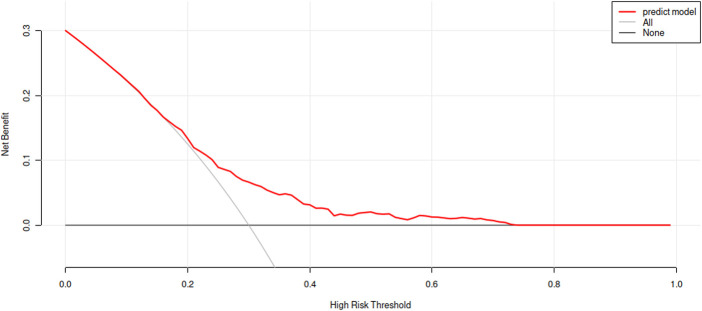
Clinical decision curve analysis of the training set.

Clinical decision curve describes the prediction model and observes the net benefit of intervention according to the model results. As shown in Figure 4, the “intervention for all” in the curve was for all people to intervene. Only positive people could benefit, with the increase of threshold probability the net benefit will change from large to small. The “intervention for none” in the curve means that no one intervenes. The net benefit of how the threshold probability changes must be zero. With the increase of threshold probability, the net benefit of the model will decrease. However, for the model, except for the small threshold probability, the model performs well in other threshold probability cases according to the prediction results of the model. Therefore, the prediction results of this model are good and can be used in clinical research.

### 3.5. The best boundary value of independent risk factors in the prediction nomogram

**Figure 5:**
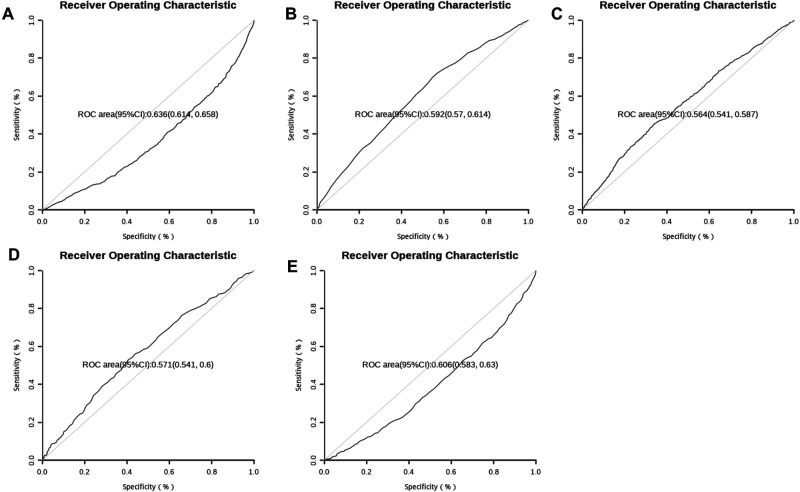
ROC curve of 5 risk factors (A:Albumin, B:Age, C:Glucose, D:Free fatty acids, E:ApoA1).

This model used R 4.0.3 statistical software for statistical analysis. The pROC package was used for ROC analysis and drawing curve. Bootstrap was used to calculate the standard error and 95% confidence interval of AUC, and wilcox rank sum test was used to compare the difference between the positive group and the negative group. The point with the largest sum of sensitivity and specificity was selected as the best boundary value as shown in Figure 5. The best boundary value of age was 72 years, the best boundary value of free fatty acid was 0.555, the best boundary value of apolipoprotein A1was 1.245, the best boundary value of glucose was 7.465, and the best boundary value of albumin was 38.895. The best boundary value can be used to evaluate independent risk factors sensitively and quantitatively.

## 4. Discussion

The metabolism of heart is aerobic. The metabolism of myocardial energy substances is produced by glycolysis (anaerobic pathway) and oxidative phosphorylation (aerobic pathway). Under physiological conditions, 90% of ATP in adults is produced by oxidative phosphorylation. During fetal heart development, the heart mainly depends on glycolysis to obtain energy (12). The fetal heart to have the ability to regenerate cardiac tissue through this specific metabolic mode and development program. With the development of the heart, the heart metabolism changes from glycolysis to oxidative phosphorylation during the neonatal period (13). The choice of substrates by the myocardium is not completely unconditional, and the relative utilization of various substrates by the myocardium is significantly different under different nutritional status, activity status and endocrine balance conditions. Myocardial ischemia makes the imbalance between oxygen supply and oxygen consumption of myocardial cells, resulting in a decrease of ATP production in mitochondria, an increase of anaerobic glycolysis of glucose, accumulation of lactic acid and fatty acid, and cell poisoning. Glycolysis becomes the main way to produce ATP at the time of myocardial ischemia. The energy generated is at 5.6 percent of the aerobic metabolic capacity by glycolysis, but it is of great significance to maintain the integrity of the myocardial cell membrane system in the event of sudden myocardial ischemia and hypoxia. The threshold of glucose uptake by the heart is 0.6–0.8 g/L (60–80 mg/dl). When the blood sugar is lower than this threshold, the heart will stop taking glucose. Glycolysis has certain limitations.Due to the exhausted of glucose energy substrate, glycolysis can not alleviate the lack of ATP. It was found that the metabolism of blood glucose, lipid and amino acid was disordered in the early stage of chronic myocardial ischemia in miniature pigs, and the endogenous metabolites in serum were significantly changed (14).

Myocardial pumping requires a lot of energy. Myocardial ischemia leads to metabolic disorder of energy supply such as glucose, amino acid and fatty acid. The rapid consumption of ATP in myocardial ischemia, which is one of the reasons for the significant decrease of serum glucose with the activation of glycolysis process. Proteins are hydrolyzed to amino acids for energy supply. In addition, the oxidative energy supply of glucose is impaired, which makes fat mobilization more compensatory. Triacylglycerol in adipose tissue can be hydrolyzed into fatty acid and glycerol, and fatty acid can combine with plasma protein to participate in energy metabolism. The accumulation of triacylglycerol metabolites in the body further leads to the disorder of lipid metabolism, which provides a necessary condition for the deposition of coronary atherosclerosis and lipid plaque. It can be seen that under the condition of myocardial ischemia and hypoxia, the metabolic disorder of glucose, protein and lipid is the basis for the progressive deterioration and prognosis of CHD. Abnormal expression of specific molecules such as glucose, apolipoprotein, fatty acid and albumin may become an important reference index for clinical diagnosis and treatment of CHD, and the specific combination pattern among them may be used for clinical decision analysis.

Insufficiency of myocardial ATP leads to metabolic and energy supply disorders. However, in addition to insufficient energy, this kind of supply disorder also causes the accumulation of metabolites in the myocardium due to the inability to completely discharge metabolites and damages the function and structure of the heart. In addition to the insufficient energy, the supply disorder can not completely discharge metabolites, which causes the accumulation of metabolites. Thus the function and structure of the heart are damaged. There are lipotoxicity and glucose toxicity in CHD. Lipotoxicity refers to the pathological accumulation of lipid intermediates, which may lead to cell dysfunction (15, 16). Excess plasma free fatty acids will inhibit the myocardial uptake of glucose, which promote the formation of insulin resistance and oxidative stress, and finally aggravate myocardial dysfunction (13). The above situation are related to the change of energy substrate, and the reduction of fatty acid oxidation. Mori H believed that the coronary artery calcification is high in patients with diabetes, which is related to the total plaque (17). Serum glucose is a predictor of outcomes of CHD, and it can evaluate the multi vessel damage (18). The study showed that albumin could improve blood flow and glucose utilization in cerebral infarction (19). Therefore, it is particularly important to explore the changes of cardiac energy metabolism substrate in MACE.

In this study, the clinical data and survival information of 5,935 patients with CHD were extracted from the medical record data of Guang'anmen Hospital. Five risk factors of MACE during hospitalization, including age, ALB, GLU, FFA, ApoA1 were determined by univariate and multivariate logistic regression models, in order to establish a prognostic nomogram. The nomogram is a prognostic tool that can predict clinical events by integrating potential risk factors. The nomogram was effectively used to predict cardiac risk factor. As far as we know, this is the first model to study the relationship between cardiac energy metabolism substrate and prognosis of MACE during hospitalization. Through the evaluation of calibration curve and clinical decision curve, satisfactory results were obtained in training set and verification set. Therefore, we established a simple and easy to obtain model to quickly identify hospitalized CHD patients, and the nomogram can be used to guide clinical practice. Compared with the existing model, the acceptable AUC of this nomogram model was 0.655. The nomogram of this study used five factors, which could be collected within 24 h of hospitalization, and performed well in the MACE during hospitalization.

We used the univariate logistic regression to find out the predictive factors, and established the model in multivariate logistic regression. The AUC (C index) under the ROC curve of the training set was 0.655 [0.621,0.689], and the AUC (C index) under the ROC curve of the validation set was 0.674 [0.623,0.724]. Calibration analysis performed in both training set and validation set showed similar probabilities, with no statistical difference between the two groups in MACE. The ROC curve of the five predictors was analyzed. The point with the largest sum of sensitivity and specificity was selected as the boundary value, and the accuracy at this boundary value were calculated. The boundary values of the five predictors are as follows: the age was 72, the free fatty acid was 0.555, the ApoA1 was 1.245, the GLU was 7.465, and the ALB was 38.895. The the boundary value can quantitatively evaluate the risk of independent factors, and pay attention to the hospital MACE of patients timely when reaching the boundary value. It can also assess the sensitivity, specificity and accuracy of risk factors in this model. Our data emphasize the importance of ALB, GLU and lipid metabolism in the prognosis of CHD. This is consistent with recent research results (20–22), which confirms the important role of energy metabolism substrate in the occurrence and development of CHD (23).

Among the five prognostic factors we used, age is considered to be a risk factor for cardiovascular disease. The decreased expression of antioxidant factors in elderly patients is a risk factor for atherosclerosis, causing lipid metabolism disorder and plaque formation, and ultimately leading to the aggravation of CHD. FFA is the main substrate for heart energy supply, and apolipoprotein is an important factor for lipid transport (24). Although nuclear magnetic resonance and other means can be used to measure the energy supply of the heart, a convenient way may be more needed in clinical practice to judge the changes in the energy metabolism substrates. In brief, we need a method to identify the occurrence of hospital MACE according to the energy supply, so as to make better clinical decisions. This is the first time to establish a prediction model of CHD based on myocardial energy metabolism substrate.

According to Table 4 below, it can be seen that the data baseline of this study is 9.3% for the youth group (18–65 years old), 14.3% for the middle-aged group (66–79 years old), and 15.8% for the elderly group (80–99 years old) according to international age segmentation standards. It was found that as the age group increased, the probability of MACE increased, and the differences between groups were statistically significant (*P* < 0.01). Albumin was divided into normal and hypoalbuminemia, and hypoalbuminemia was classified into three levels: mild, moderate, and severe. It was found that the probability of MACE in the hypoalbuminemia group was higher than that in the normal albumin group, and the probability of MACE increased with the increase of the severity of hypoalbuminemia. The difference between groups was statistically significant (*P* < 0.01). Glucose refers to the fasting venous glucose content, which was divided into normal blood glucose levels below 6.1 mmol/L, abnormal glucose tolerance levels between 6.1 and 7.0, high blood glucose levels between 7.0 and 13.9, and high blood glucose levels above 13.9 that require insulin activation. When blood glucose was less than 6.1, the probability of MACE was higher, and the energy substrate was insufficient at this time. There was no statistically significant difference between 6.1 and 7.0. Blood glucose levels above 13.9 MACE are lower, providing sufficient energy for the myocardium. Free fatty acids were divided into low free fatty acids under 0.3, normal values between 0.3 and 0.9, and high free fatty acid groups above 0.9. When coronary heart disease occured, the accumulation of free fatty acids should be reduced. Although 0.3 to 0.9 is normal, it does not seem to provide a substrate for myocardial metabolism. It does not seem to reduce lipid accumulation when it was less than 0.3, so the benefit was minimal at normal values. Apolipoprotein A1 was divided into low apolipoprotein A1 < 1, normal values of 1.0–1.6, and the values of greater than 1.6. When apolipoprotein A1 < 1, the ability to transport lipids decreased, and the probability of MACE was higher, with statistical significance (*P* < 0.05) compared to the group > 1.6. Therefore, selecting these 5 factors as model variables can benefit within the range of grouped variables.

**Table 4:** Comparison of data baseline between major adverse cardiovascular events (MACE) group and non-MACE group.

Variables	Sum	*¯x *± SD	Minimum	Maximum	MACE group	MACE and non-MACE *P* value
**Age, years (24–97)**						
Age group	3,028	72.51 ± 11.77	24.00	119.0	425 (14%)	<0.0001
Underage group (0–17)	0	0	0	0	0	0
Youth group (18–65)	876	57.42 ± 6.730	24.00	65.00	82 (9.3%)	<0.0001
Middle-aged group (66–79)	1,147	73.59 ± 4.122	66.00	79.00	165** (14.3%)	<0.0001
Elderly group (80–99)	1,005	84.44 ± 4.039	80.00	97	178** (15.8%)	<0.0001
**Albumin, g/L (21.6–54.2)**						
Albumin group	3,028	39.06 ± 5.010	21.60	54.20	425 (14%)	0.0008
Hypoalbuminemia group (<35)	589	31.52 ± 2.720	21.60	34.95	150 (13%)	0.0148
Mild hypoalbuminemia group (30–35)	447	32.79 ± 1.330	30.00	34.95	101 (22.6%)	<0.0001
Moderate hypoalbuminemia group (25–30)	125	28.10 ± 1.313	25.10	29.90	40^##^ (32.2%)	0.9230
Severe hypoalbuminemia group (<25)	17	23.31 ± 0.9549	21.60	24.80	7^##^ (43.7%)	0.7064
Normal albumin group (35–55)	2,439	40.88 ± 3.511	35.00	54.20	274** (11.2%)	0.0007
**Glucose, mmol/L (0.64–33.4)**						
Glucose group	3,018	7.835 ± 3.507	0.6400	33.40	425 (14%)	<0.0001
<6.1	1,132	5.244 ± 0.5661	2	6.090	129 (5.2%)	<0.0001
6.1–7.0	486	6.503 ± 0.2583	6.100	6.990	66 (2.7%)	0.9903
7–13.9	1,223	9.214 ± 1.843	7.000	13.84	192** (7.8%)	0.0051
>13.9	187	17.97 ± 3.807	13.90	33.40	37** (1.5%)	<0.0001
**Free fatty acid, umol/L (0.03–2.35)**						
Free fatty acid group	3,028	0.5590 ± 0.2899	0.03000	2.350	425 (14%)	0.0047
<0.3	487	0.2140 ± 0.05963	0.03000	0.2900	53 (2.1%)	<0.0001
0.3–0.9	2,197	0.5441 ± 0.1569	0.3000	0.8900	307** (12.5%)	0.0045
>0.9	344	1.143 ± 0.2590	0.9000	2.350	65** (2.6%)	<0.0001
**Apolipoprotein A1, g/L (0.34–2.43)**						
Apolipoprotein A1 group	3,028	1.144 ± 0.2421	0.3400	2.430	425 (14%)	<0.0001
<1	850	0.8672 ± 0.1071	0.3400	0.9900	165 (6.7%)	<0.0001
1–1.6	2,075	1.228 ± 0.1513	1.000	1.590	254* (10.4%)	0.0427
>1.6	103	1.752 ± 0.1584	1.600	2.430	6 (0.2%)	0.0840

The results were expressed as mean ± standard deviation, or percentage. **For comparison with the first group between groups *P* < 0.01, and * for comparison with the first group between groups *P* < 0.05. ^##^ was compared to the first group within the group *P* < 0.01, and ^#^ was compared to the first group within the group *P* < 0.05.

The grouping criteria were as follows. The age was divided into four levels based on international age group classification standards. Albumin was divided into normal and hypoalbuminemia, and hypoalbuminemia was classified into three levels: mild, moderate, and severe. Glucose was divided into normal blood glucose levels below 6.1, abnormal glucose tolerance levels between 6.1–7, high blood glucose levels between 7–13.9, and high blood glucose levels above 13.9 that require insulin activation. Free fatty acids were divided into low free fatty acids below 0.3, normal values between 0.3–0.9, and high free fatty acids above 0.9. Apolipoprotein A1 was divided into low apolipoprotein A1 < 1, normal values of 1–1.6, and normal values of >1.6.

This study still has shortcomings: If the model has too many prediction factors, the accuracy of the model will be affected by over fitting. Therefore, our study selected five factors. The myocardial infarction thrombolysis (TIMI) score, the global acute coronary event registry (GRACE) score, and the HEART score are commonly used tools to predict the short-term and long-term outcomes in acute myocardial infarction (25–29). Because those scores cannot be obtained, the nomogram model cannot be compared with those scoring models (30). We can not perform time dependence analysis because some parameters such as glucose and blood lipid change. We only collected the observations at one time point. The statistical results may be biased because we did not fully consider the treatment of diabetes, hyperlipidemia, hypertension, heart failure and other results. The model still needs more samples to verify its feasibility, and the model was constructed using data from Beijing. Our patients are Asian, so it is necessary to verify our model in different populations.

Some studies point out that metabolic regulation therapy may be effective, especially when it is aimed at restoring the fuel balance of fatty acid-glucose-albumin. But patients of heart failure will also take a series of drugs that may affect heart metabolism. Therefore, it is very important to establish a non-invasive model of cardiac metabolism. The boundary value can guide the strategy of metabolic therapy. Therefore, our study developed a prognostic nomogram. The association of ALB, GLU, lipid metabolism and prognosis of CHD was established well to predict MACE during hospitalization. The independent predictive ability of age, ALB, GLU, APOA1 and FFA were quantified, which can be used for the evaluation and treatment of CHD patients receiving clinical treatment. This model establish the best boundary values of risk factors. The independent predictive ability of risk factors can be used for the evaluation and treatment of CHD patients.

## 5. Conclusion

In summary, age, ALB, FFA, ApoA1 and GLU are independent factors for MACE during hospitalization in CHD patients, and a nomogram model for MACE during hospitalization risk prediction in CHD patients constructed based on the above factors has good discrimination, calibration,and clinical effectiveness and can be used as an effective tool for early clinical prediction of in-hospital MACE risk in CHD patients. The ROC curve is used to establish the best boundary value of the five risk factors, which can quantitatively present the changes of cardiac energy metabolism substrate, and finally achieve prediction of MACE during hospitalization conveniently and sensitively.

## Data Availability

The raw data supporting the conclusions of this article will be made available by the authors, without undue reservation.
